# Digital technique to analyze the wear of the slot after orthodontic treatment through fixed multibracket appliances

**DOI:** 10.1186/s12903-023-02818-1

**Published:** 2023-03-14

**Authors:** Mabel Daiana Pimentel-García, Álvaro Zubizarreta-Macho, Jorge Alonso Pérez-Barquero, Clara Guinot Barona, Alberto Albaladejo Martínez

**Affiliations:** 1 Department of Orthodontics, Faculty of Medicine and Dentistry, University of Salamanca, Paseo Universidad de Coimbra, 37008 Salamanca, Spain; 2 Department of Endodontics, Faculty of Health Sciences, Alfonso X El Sabio University, 28691 Madrid, Spain; 3 Department of Stomatology, Faculty of Medicine and Dentistry, University of Valencia, 46010 Valencia, Spain; 4 Department of Dentistry, Faculty of Medicine and Health Sciencies, Catholic University of Valencia, 46001 Valencia, Spain

**Keywords:** Micro-computed tomography, SEM, Bracket slot, Orthodontics, Wear, Fixed multibracket appliance

## Abstract

**Introduction:**

To assess the accuracy, repeatability and reproducibility of a measurement digital technique to quantify the wear of the bracket slot walls of the fixed multibracket appliance after orthodontic treatment with the previous measurement traditional technique (scanning electronic microscope (SEM)).

**Methods:**

A total of 100 fixed multibracket appliances were cemented during the 15 months orthodontic treatment and subsequently removed. The fixed multibracket appliances were submitted preoperatively and postoperatively to a micro-computed tomography (micro-CT) scan to obtain accurate standard tessellation language (STL) digital files of the fixed multibracket appliances and to a preoperatively and postoperatively SEM analysis. Afterwards, pre-operatively and postoperatively STL digital files of each fixed multibracket appliances were aligned using morphometric software with the best fit algorithm. Subsequently, area and volume wear of fixed multibracket appliances was identified, isolated and measured.

**Results:**

The repeatability and reproducibility of the digital measurement method for the area (mm^2^) and volume (mm^3^) were analyzed by Gage R&R statistical analysis. The area wear of the bracket slot walls of the fixed multibracket appliance after orthodontic treatment showed a repeatability of 3.7% and a reproducibility of 0%. The volume of the bracket slot walls of the fixed multibracket appliance after orthodontic treatment showed a repeatability of 0.9% and a reproducibility of 5.6%. However, the traditional measurement technique showed a repeatability of 0.58% and a reproducibility of 33.01%; hence, it was repeatable but not reproducible.

**Conclusions:**

The digital measurement technique is a reproducible, repeatable, and accurate method for quantifying the wear of the bracket slot walls of the fixed multibracket appliance after orthodontic treatment.

## Background

Fixed multibracket appliances have been widely used to perform orthodontic treatments, enabling the tooth movement by transferring the forces from the archwire to the teeth [1]. The root position after orthodontic treatment is the result of torquing movements generated by an orthodontic archwire inside the bracket slot, specifically between the archwire and the bracket slot walls [1]. Torque is determined by the tooth inclination, slot-wire play, and the multibracket appliance material [2]; therefore, researchers have highlighted the impact of slot dimension on torque expression [3, 4]. Moreover, McKnight et al. did not recommend full thickness stainless steel rectangular arch wires with stainless steel multibracket appliances, to prevent large deformation of the brackets by overloading [5]; however, the continued forces generated during the orthodontic treatment between both metallic structures might lead the wear of the bracket slot, and hence affect to the dental movement generated by the fixed multibracket appliance. Therefore, several studies have been conducted to analyze the impact of torsional forces on the predictability of tooth movement [6, 7].

Design variations on the profile of the bracket slot may have an impact on torque expression of the incisors which is highly relevant for the final aesthetic result and for the frictional resistance which may determinate the force required for moving teeth [8, 9]. In addition, some reports have evidenced the impact of the slot dimensions torque expression and torque play [10, 11]. Previous research has been conducted to analyze the wear and deformation of dental materials [1, 5, 10, 12–17]; However, other studies have evaluated the changes occurred in the bracket slot using different measurement techniques, like scanning electron micrographs [18, 19], digital microscope [5] and computer-aided light microscopy [1]. Additionally, scanning electron microscopy (SEM) has been used in studies of orthodontics [20], implant [21], and lingual orthodontics [22], however, these studies required extensive instrumentation, time and did not allow microscale measurements. In addition, frictional resistance between bracket slot and orthodontic archwire has been also measured and compared among different materials as: stainless steel, titanium, ceramic, and plastic brackets [23, 24], allowing analyzing the impact of the geometry and material of the bracket slot on the frictional resistance, although did not allow measuring accurately the area and volume of the wear produced in the bracket slot walls. Therefore, a new technique for measuring accurately the area and volume of the bracket slot is necessary, to provide reliable data for future research.

The aim of this work was to analyse and compare a measurement digital technique with the previous measurement traditional technique (SEM) to quantify the wear of the bracket slot walls of the fixed multibracket appliance after orthodontic treatment, with a null hypothesis (H_0_) stating that each technique provides repeatable and reproducible measures to quantify the wear of the bracket slot walls of the fixed multibracket appliance after orthodontic treatment.

## Methods

### Study design

This experimental study was conducted in the Master’s degree in Orthodontics at University of Salamanca (Salamanca, Spain), and the Department of Stomatology at University of Valencia (Valencia, Spain), between November 2020 and Junio 2022 in accordance with the ethical guidelines established by the Declaration of Helsinki [25] and the CONSORT Statement [26] and was authorized by the Ethical Committee of the Faculty of Health Sciences, University of Salamanca (Salamanca, Spain), in December 2020 (process no. 19/2020). All patients gave their informed consent to provide the digital files.

### Clinical procedure

**Fig. 1:**
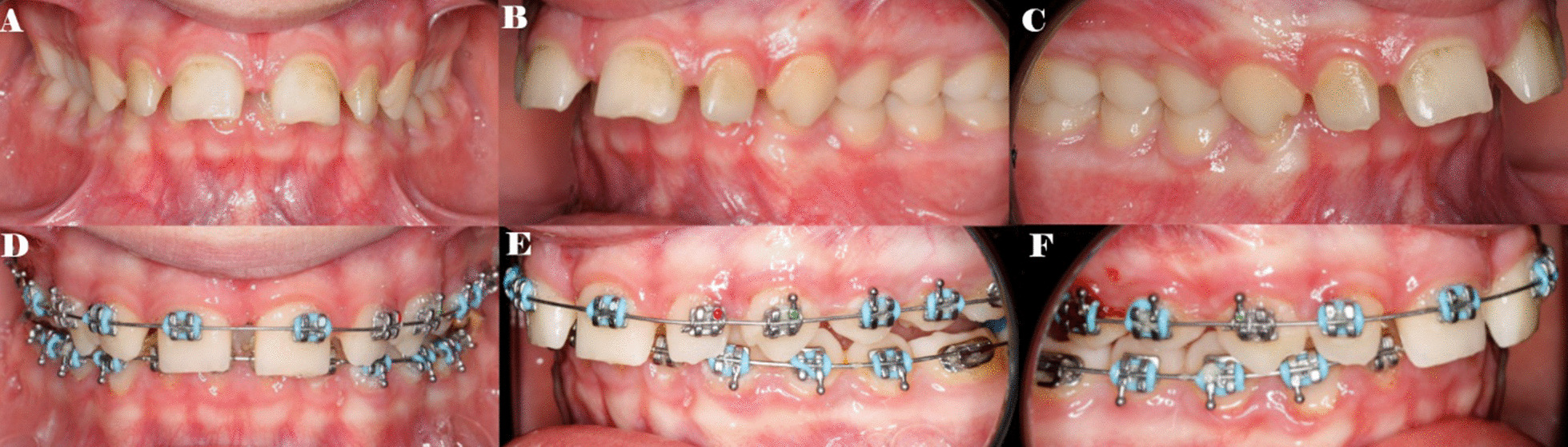
**A** Preoperative intraoral images of the frontal, **B** left lateral and **C** right lateral and **D** postoperative intraoral images of the frontal, **E** left lateral and **F** right lateral with the fixed multibracket appliances

One hundred multibracket appliances (3GbracketsSCAPE .022MBT, Pacific Orthodontics, Guadalajara, Spain) were cemented from tooth 1.5 to tooth 2.5, in the center of the buccal surface of the clinical crown with a photo-polymerized composite resin cement (Transbond™ XT, 3 M ESPE™, Saint Paul, MN, USA) before etching the enamel buccal surface with 37% orthophosphoric acid (VOCOCID, VOCO GmbH, Cuxhaven, Germany) for 20 s and photo-polymerized resin adhesive primer application (Unitek Transbond™ XT, 3 M ESPE™, Saint Paul, MN, USA) for 20 s (Fig. 1). A sequence of NiTi alloy archwires were used in both upper and lower arches with 0.16 Niti, 0.18 Niti, 16 × 22 Niti alloy archwires, and it was finished with an stainless steel alloy 19 × 25 archwire during the 15 months orthodontic treatment, including crossbite and intercuspation elastics and chains; since the selected patients presented a diagnoses of Classes II division I, positive dental bone discrepancy in the upper arch, pro-inclination of the upper incisors, and retro-inclination of the lower incisors. Afterwards, the fixed multibracket appliances (3GbracketsSCAPE .022MBT, Pacific Orthodontics, Guadalajara, Spain) were removed from tooth 1.5 to tooth 2.5 with a specific instrument to remove the fixed multibracket appliances (3GbracketsSCAPE .022MBT, Pacific Orthodontics, Guadalajara, Spain). All orthodontic treatments; including debonding procedures, were performed by an unique operator with 10 years’ experience.

### Experimental procedure

#### Digital measurement technique using micro-CT scanning procedure

**Fig. 2:**
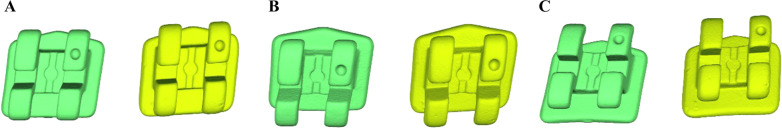
**A** Frontal view, **B** top view and **C** bottom view of the STL1 (green) and STL2 (yellow) of the bracket of position 2.1

All of the fixed multibracket appliances (3GbracketsSCAPE .022MBT, Pacific Orthodontics, Guadalajara, Spain) were submitted twice to a micro-Computed Tomography (micro-CT) scan (Skyscan 1176, Bruker-MicroCT, Kontich) with the following exposure parameters: 160.0 kilovolt peak, 56.0–58.0 microamperes, 500.0 ms, 720 projections, 4 frames, a tungsten target between 0.25 and 0.375 mm, a 3 µm resolution, and a pixel size of 0.127 µm, to obtain accurate Standard Tessellation Language (STL) digital files of the fixed multibracket appliances (3GbracketsSCAPE .022MBT, Pacific Orthodontics, Guadalajara, Spain). The first micro-CT scan (Micro-CAT II, Siemens Preclinical Solutions, Knoxville, TN, USA) was performed before the orthodontic treatment through fixed multibracket appliances (3GbracketsSCAPE .022MBT, Pacific Orthodontics, Guadalajara, Spain) (Fig. 2) (STL1) and the second micro-CT scan (Micro-CAT II, Siemens Preclinical Solutions, Knoxville, TN, USA) was performed after the orthodontic treatment through fixed multibracket appliances (3GbracketsSCAPE .022MBT, Pacific Orthodontics, Guadalajara, Spain) (Fig. 2) (STL2).

#### Alignment procedure

**Fig. 3:**
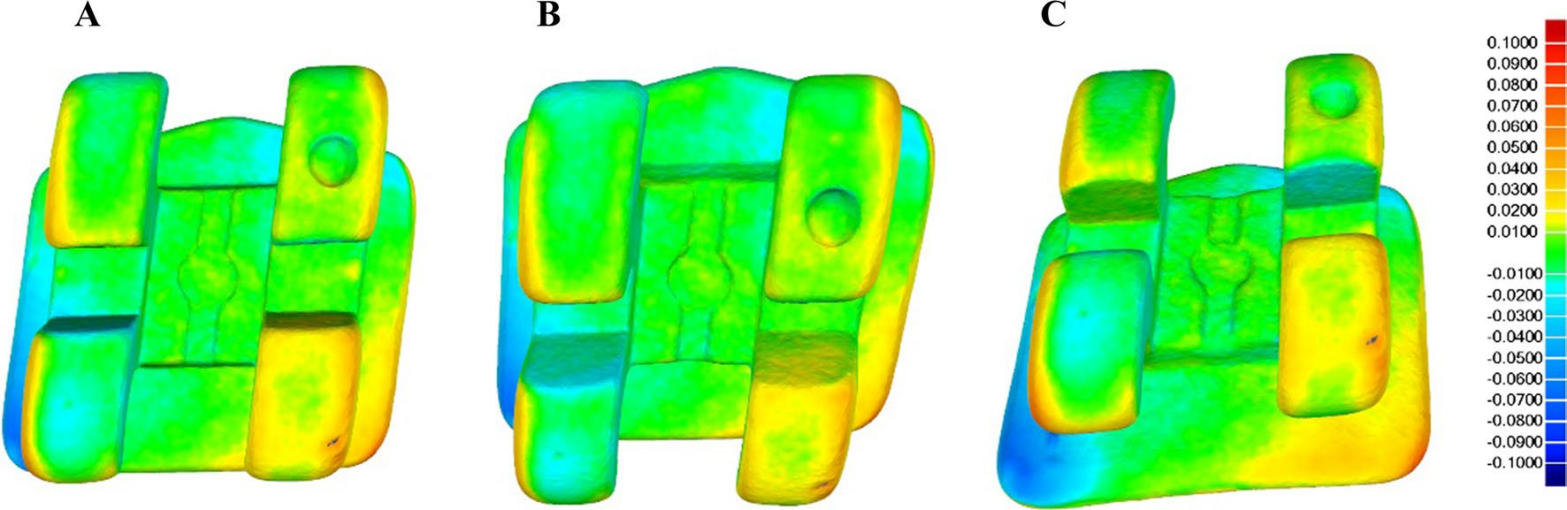
**A** Frontal view, **B** top view and **C** bottom view of the alignment procedure between STL1 and STL2 of the bracket of position 2.1 and the spectrum values. Warm colours represent a volume increase, cold colours represent a volume decrease, and green represents an accurate alignment

Once the preoperative (STL1) and postoperative (STL2) micro-CT scans (Skyscan 1176, Bruker-MicroCT, Kontich) of the fixed multibracket appliances (3GbracketsSCAPE .022MBT, Pacific Orthodontics, Guadalajara, Spain) were uploaded to a reverse engineering geomorphometric software (3D Geomagic Capture Wrap, 3D Systems^©^, Rock Hill, SC, USA) and an alignment procedure of the STL digital files was done with the best fit algorithm (Fig. 3).

#### Measurement procedure

**Fig. 4:**
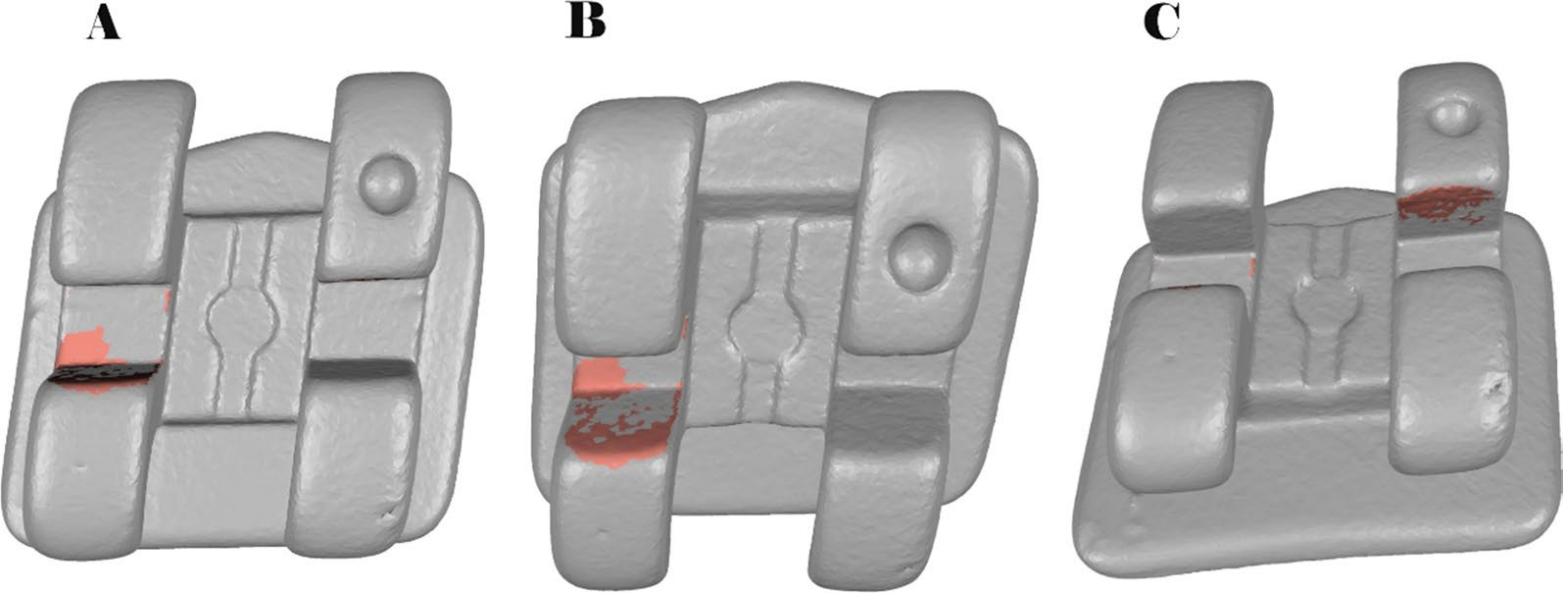
**A** Frontal view, **B** top view and **C** bottom view of the wear located at the bracket slot walls

Afterwards, the area and volume differences between STL1 and STL2 were analyzed. The spectrum between the alignment of STL1 and STL2 digital files was set at ± 100 µm and the tolerance at ± 10 µm (Fig. 4).

In addition, area measurement was performed between STL1 and STL2 to determine the wear of the fixed multibracket appliances (3GbracketsSCAPE 0.022MBT, Pacific Orthodontics, Guadalajara, Spain) in area and volume by the digital measurement technique.

### Traditional measurement technique using scanning electron microscopy procedure

The fixed multibracket appliances were preoperatively and postoperatively exposed to a SEM (HITACHI S-4800, Fukuoka, Japan) at 30 × and 600 × magnification using the following exposure parameters: 20 kV acceleration voltage, resolution ranging from − 1.0 nm at 15 kV to 2.0 nm at 1 kV, and magnification from 100 to 6500 × .

#### Measurement procedure

**Fig. 5:**
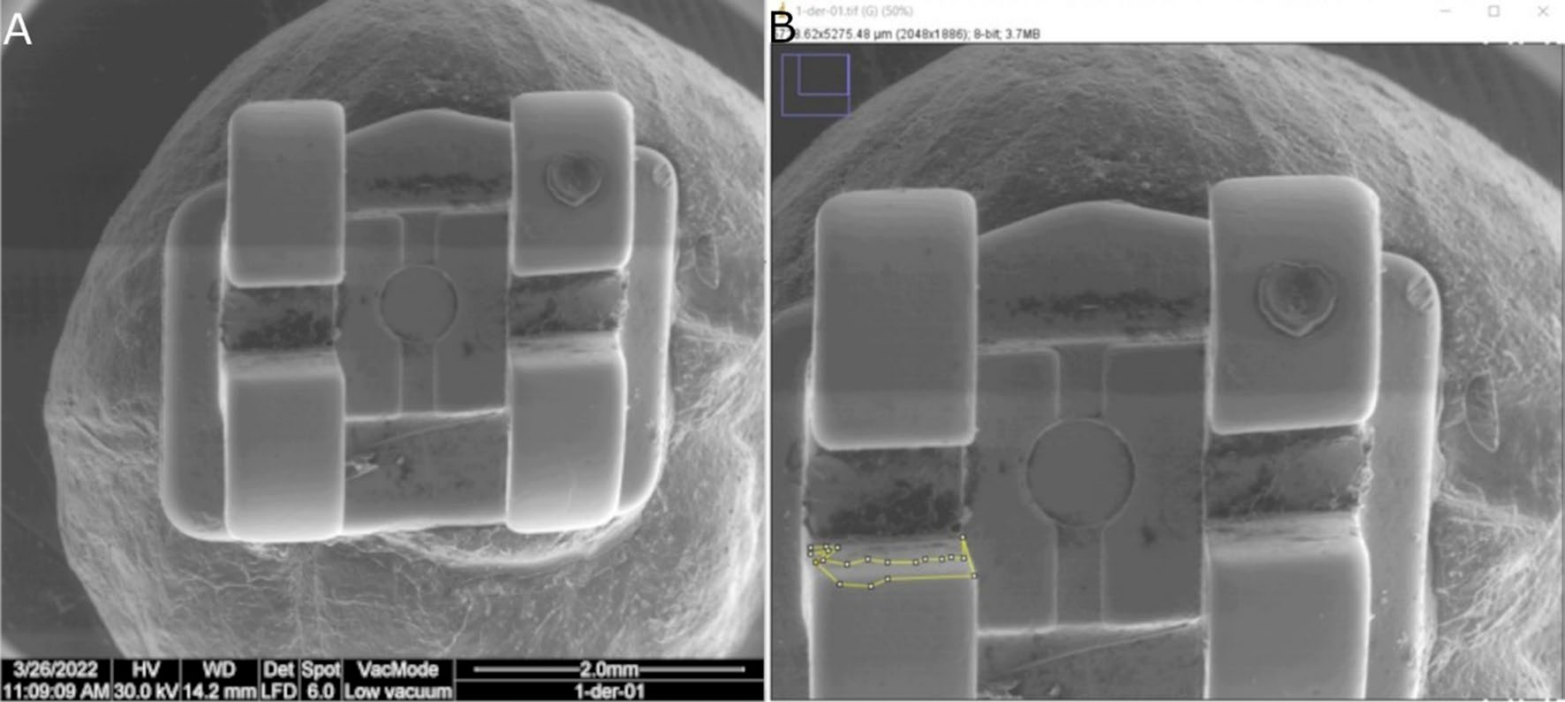
SEM image (**A**), area selection to measurement the wear (**B**)

Afterwards, the images obtained from the SEM analysis were uploaded into an image management software (NIH ImageJ Software) (Fig. 5A) to analyze the wear of the slot of the fixed multibracket appliances (Fig. 5B) [20].

### Validation of repeatability and reproducibility of the technique

In order to validate the repeatability and reproducibility of the digital measurement technique and the traditional measurement technique to quantify the wear of the slot of the fixed multibracket appliance after orthodontic treatment, the measures were repeated twice by two operators (Operator A and Operator B) and Gage R&R statistical analysis was performed.

### Statistical tests

The variables of interest were registered for statistical analysis (SPSS 22.00, Microsoft inc, Redmond, WA, USA) and R (R Foundation for Statistical Computing, Vienna, Austria). The repeatability and reproducibility of the digital measurement method for the area (mm^2^) and volume (mm^3^) were analyzed by Gage R&R statistical analysis. In addition, the repeatability and reproducibility of the traditional measurement method for the area (mm^2^) were also analyzed by Gage R&R statistical analysis.

## Results

### Digital measurement technique

**Fig. 6:**
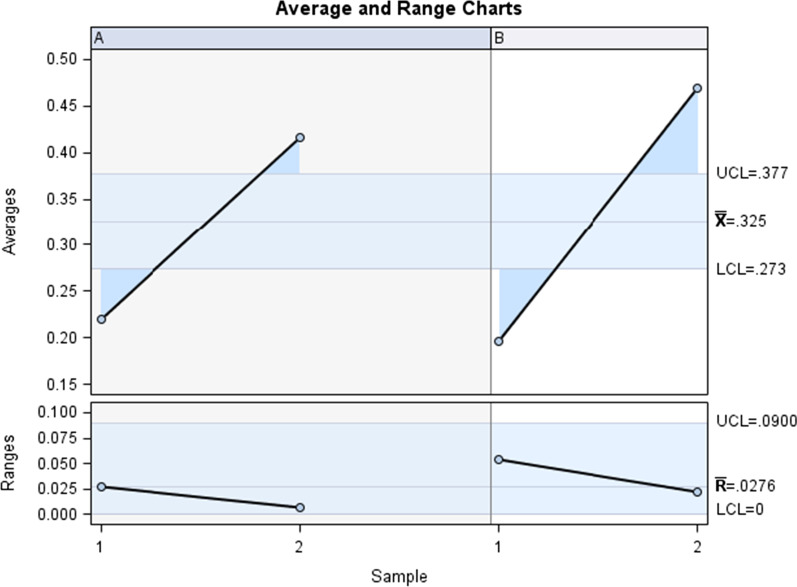
Charts for the average of the two measures of the wear area (mm^2^) of the bracket slot walls of the fixed multibracket appliance after orthodontic treatment

**Fig. 7:**
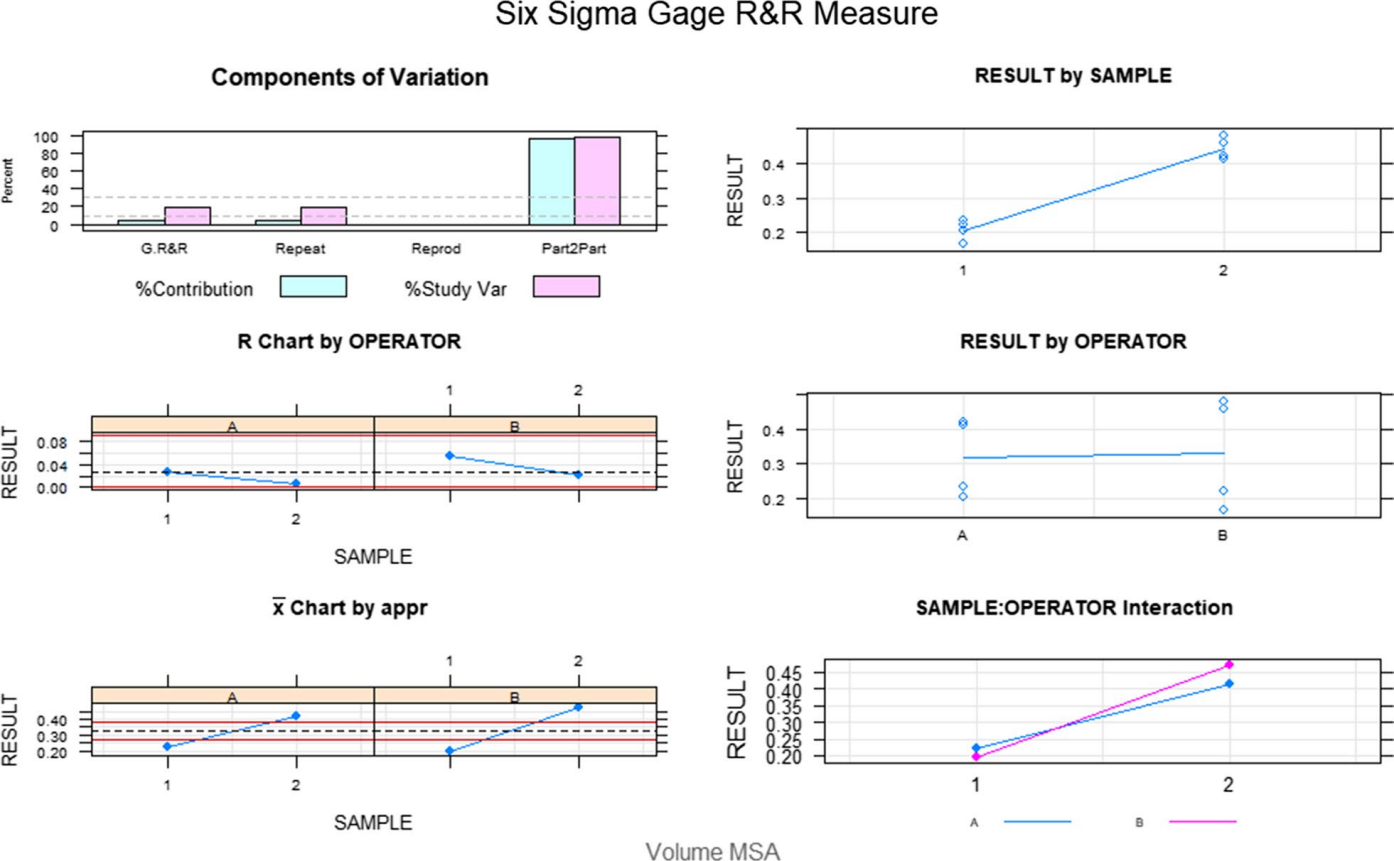
Measurement system analysis related to the wear area (mm2) of the bracket slot walls of the fixed multibracket appliance after orthodontic treatment with a chart of the contribution of each component to the total variance (Components of Variation), a mean control chart and a range control chart (R Chart by Operator and × Chart by appr), every measurement point in the graph (Trial by I and Trial by Operator), and the interactions between the operators (i): Operator interaction)

The Gage R&R statistical analysis [27, 28] of the digital measurement technique to quantify the wear area (mm^2^) of the bracket slot walls of the fixed multibracket appliance after orthodontic treatment showed that the variabilities attributable to the digital measurement technique were 3.7% (among the measures of each operator) and 0% (among the measures of the operators); respectively, of the total variability of the samples. The digital measurement technique to quantify the wear area (mm^2^) of the bracket slot walls of the fixed multibracket appliance after orthodontic treatment was considered repeatable and reproducible because the variabilities were under 10%, which is considered repeatable and reproducible (Figs. 6 and 7).

**Fig. 8:**
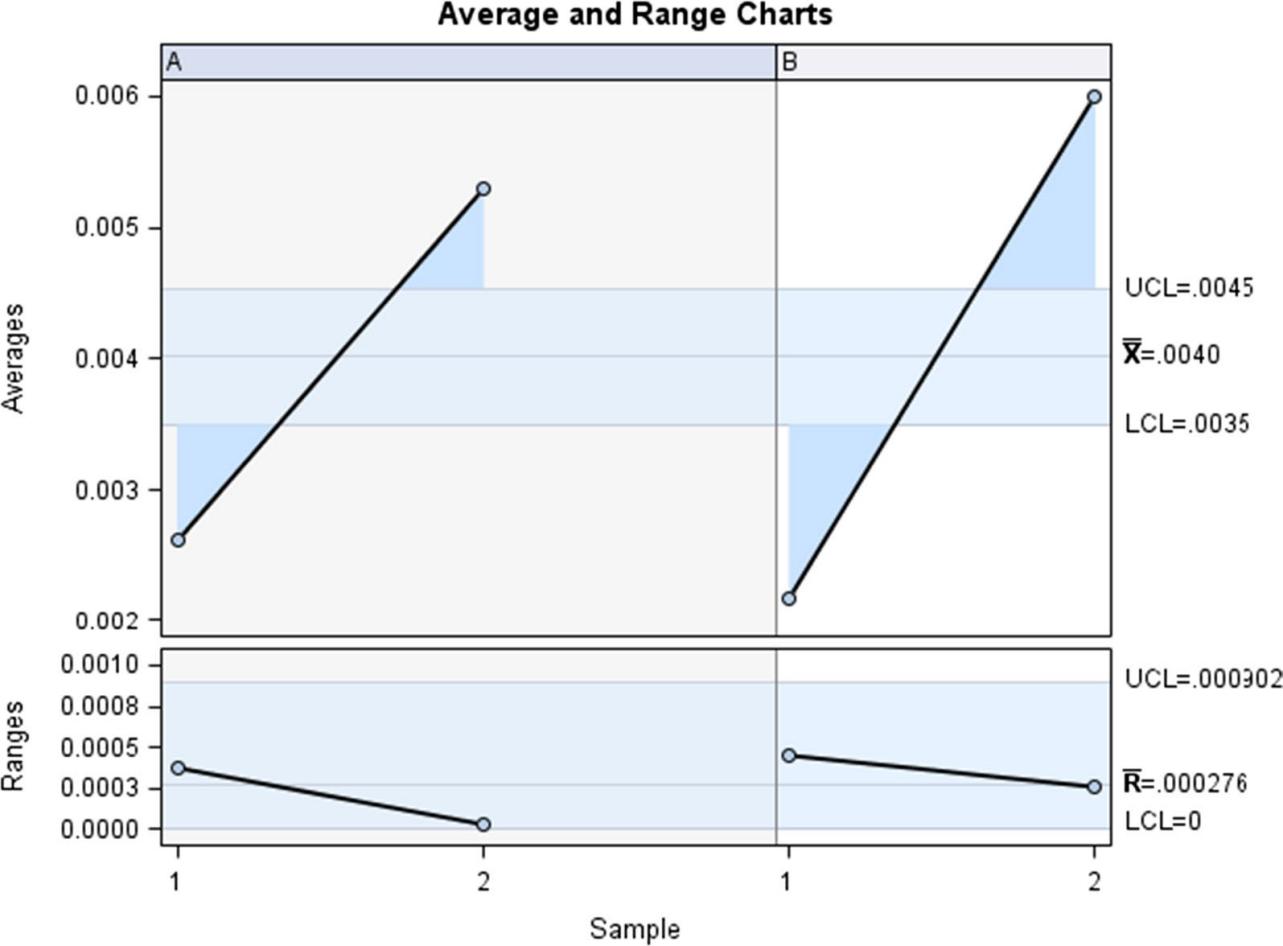
Charts for the average of the two measures of the wear volume (mm3) of the bracket slot walls of the fixed multibracket appliance after orthodontic treatment

**Fig. 9:**
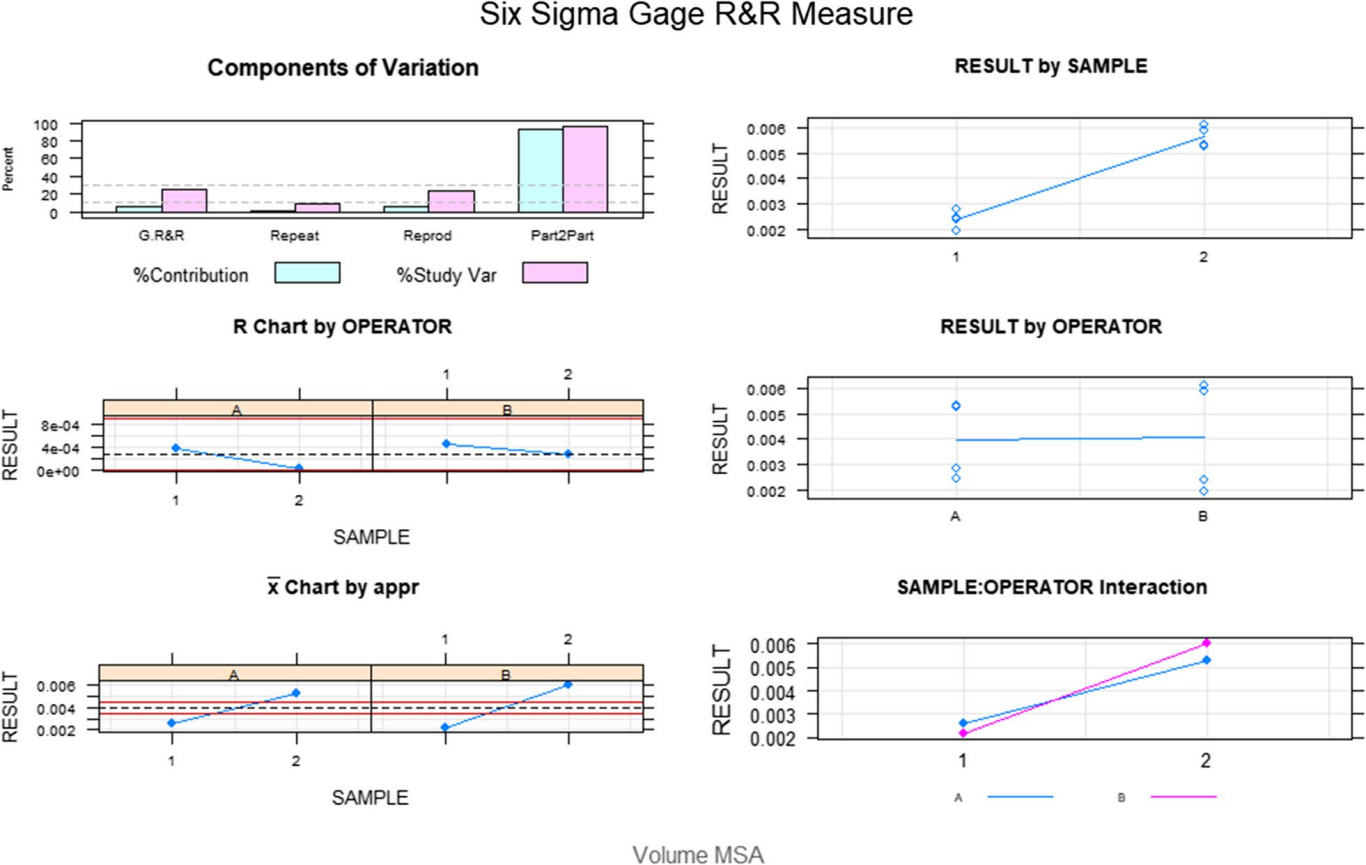
Measurement system analysis related to the wear volume (mm^3^) of the bracket slot walls of the fixed multibracket appliance after orthodontic treatment with a chart of the contribution of each component to the total variance (Components of Variation), a mean control chart and a range control chart (R Chart by Operator and × Chart by appr), every measurement point in the graph (Trial by I and Trial by Operator II), and the interactions between the operators (i): Operator interaction)

The Gage R&R statistical analysis of the digital measurement technique to quantify the wear volume (mm^3^) of the bracket slot walls of the fixed multibracket appliance after orthodontic treatment showed that the variabilities attributable to the digital measurement technique were 0.9% (among the measures of each operator) and 5.6% (among the measures of the operators); respectively, of the total variability of the samples. The digital measurement technique to quantify the wear area (mm^3^) of the bracket slot walls of the fixed multibracket appliance after orthodontic treatment was considered repeatable and reproducible because the variabilities were under 10%, which is considered repeatable and reproducible (Figs. 8 and 9).

### Traditional measurement technique

**Fig. 10:**
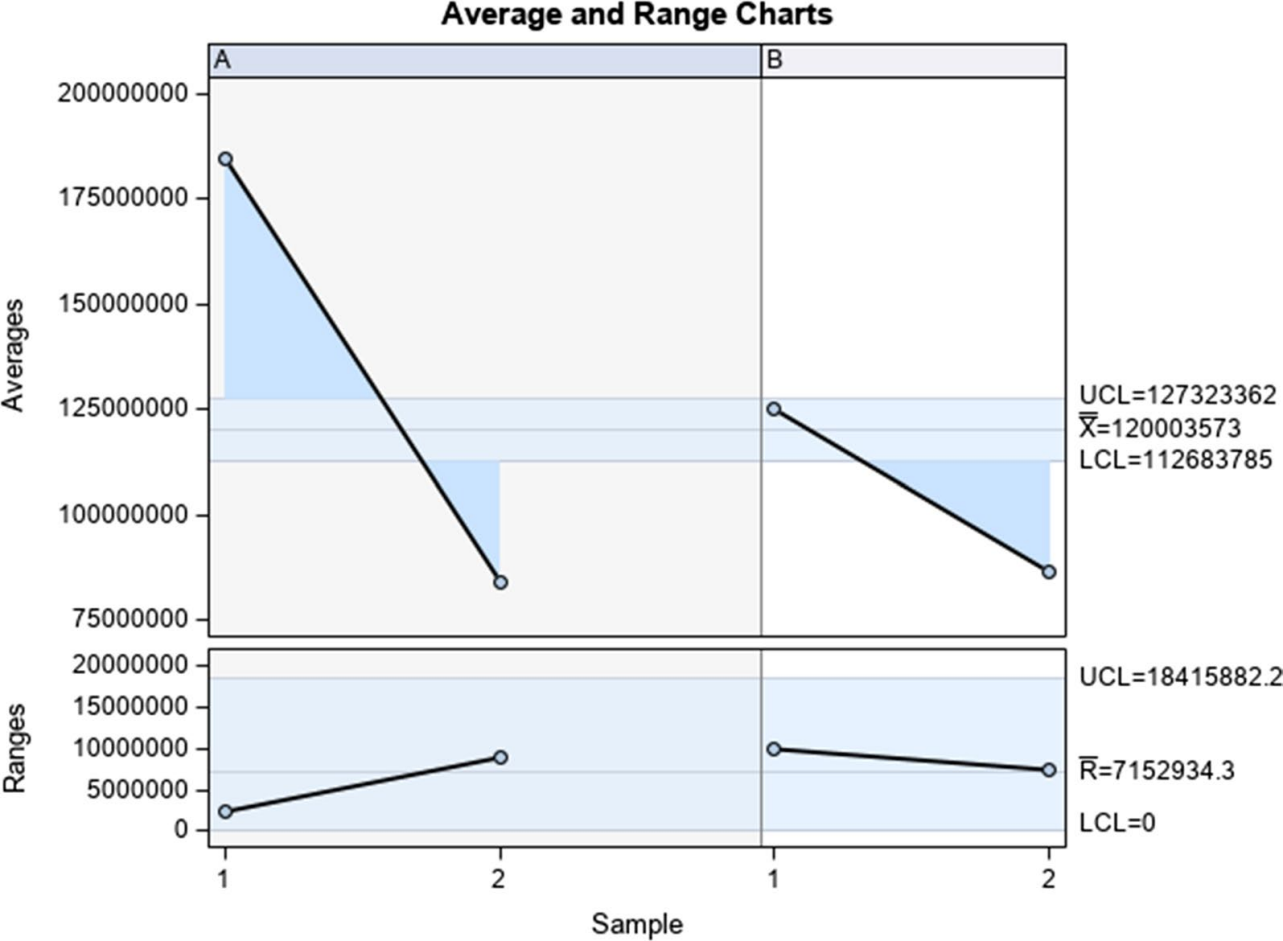
Charts for the average of the two measures of the wear volume (mm3) of the bracket slot walls of the fixed multibracket appliance after orthodontic treatment

**Fig. 11:**
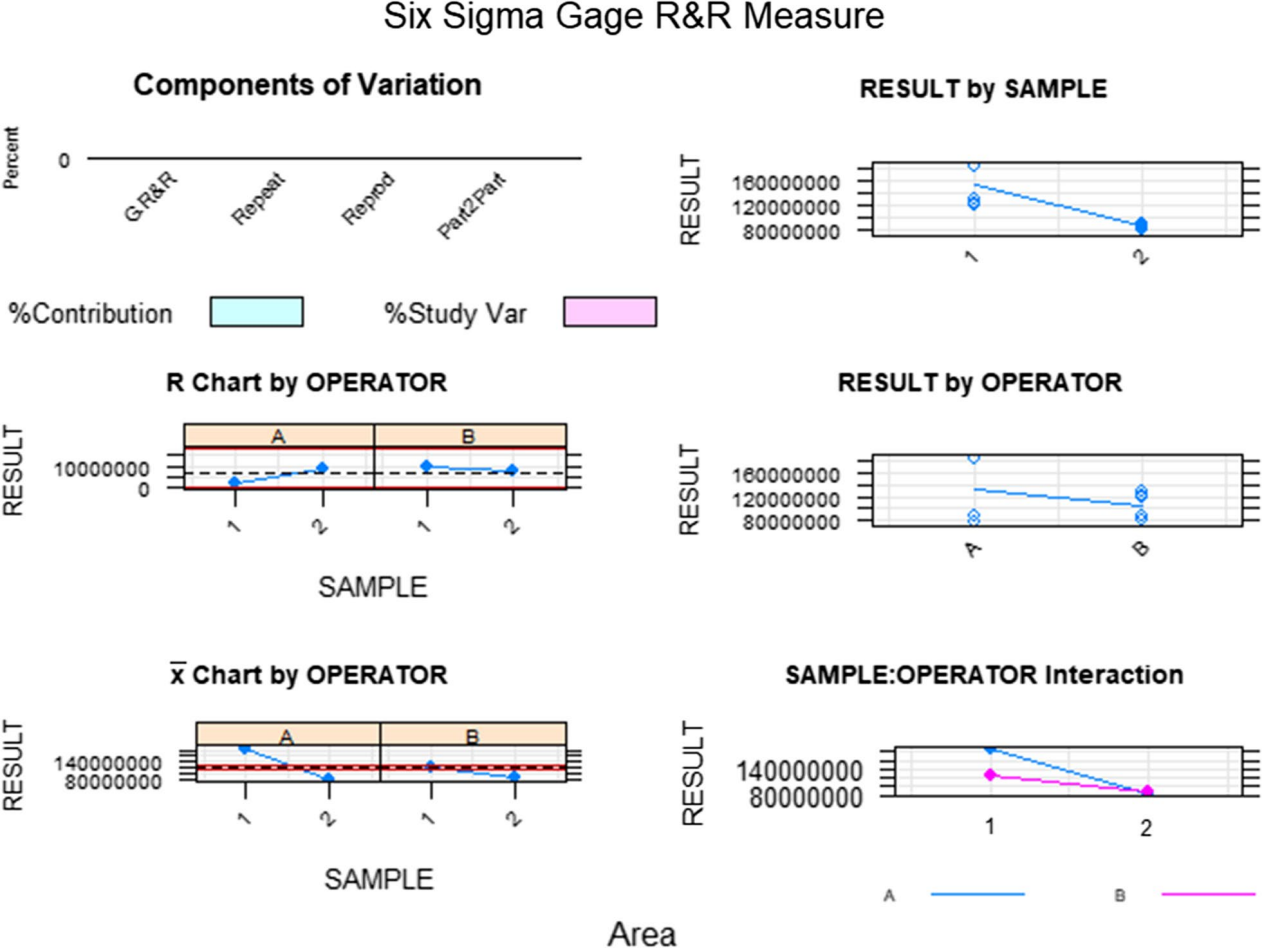
Measurement system analysis related to the wear volume (mm^3^) of the bracket slot walls of the fixed multibracket appliance after orthodontic treatment with a chart of the contribution of each component to the total variance (Components of Variation), a mean control chart and a range control chart (R Chart by Operator and × Chart by appr), every measurement point in the graph (Trial by I and Trial by Operator II), and the interactions between the operators (i): Operator interaction)

The Gage R&R statistical analysis of the digital measurement technique to quantify the wear area (mm^2^) of the bracket slot walls of the fixed multibracket appliance after orthodontic treatment showed that the variabilities attributable to the digital measurement technique were 0.58% (among the measures of each operator) and 33.01% (among the measures of the operators); respectively, of the total variability of the samples. The traditional measurement technique to quantify the wear area (mm^2^) of the bracket slot walls of the fixed multibracket appliance after orthodontic treatment was considered repeatable because the variabilities were under 10%, which is considered repeatable, but it was not reproducible (Figs. 10 and 11).

## Discussion

The results presented in this study reject the null hypothesis (H_0_) that determines that each technique provides repeatable and reproducible measures to quantify the wear of the bracket slot walls of the fixed multibracket appliance after orthodontic treatment.

Measuring the wear of the bracket slot walls of the fixed multibracket appliance after orthodontic treatment is still a challenge, since most brackets slots have rounded corners, and/or unparallel slot walls which could lead to a trapezoidal slot [21]; therefore, the digital measurement technique has proven to be useful to measure the wear of the bracket slot in both area and volume, being repeatable and reproducible. However, the traditional measurement technique showed to be repeatable but not reproducible to measure only the wear area of the bracket slot walls of the fixed multibracket appliance after orthodontic treatment.

Previous reports have highlighted the impact of slot dimensions on torque expression and torque play [14, 15], and even Kapur et al. [13] reported the influence of the deformation of the fixed multibracket appliance deformation on the anterior torque loss. Melenka et al. [23] showed that 0.038 mm stainless steel alloy fixed multibracket appliances and 0.013 mm titanium alloy fixed multibracket appliances suffered a plastic deformation; however, titanium alloy fixed multibracket appliances suffered less deformation than stainless steel alloy fixed multibracket appliances after torquing application. Therefore, some measurement techniques have been developed to analyze the slot dimensions; such as optical measurement devices, which can measure lineal distances in the slot; however, Cash et al. reported limitations in selecting repeatable reference points because of the non-orthogonal nature of the fixed multibracket appliance [10, 24].

Cash et al. [10] analyze the influence of slot height and reported that different brands of fixed multibracket appliance may have different slot shapes; furthermore, Meling et al. [4] used an indirect method to measure the slot height by measuring torque play; however, this measurement technique is not useful in fixed multibracket appliance with rectangular-shape slot. However, Major et al. [18] proposed a method of measuring slot profiles by assuming a trapezoidal slot shape. They selected 15 points on the inner profile of the brackets slot and three lines were fitted to define the slot profile. Afterwards, calculations were performed to measure a series of parameters such as slot height and slot taper.

Torquing in orthodontics is commonly used for root positioning by twisting the required segment of the rectangular archwire [29, 30]. Furthermore, torque is considered one of the most clinically demanding tooth movements; although a fraction of the torque into the bracket could remain unexpressed preventing the adjustment of the archwire into the bracket slot and thus, leading a third order clearance [31], and finally, these forces tend to deform the slot walls what can influence the predictability of tooth movement [5, 11, 32–35].

Some measurement techniques have been proposed to analyze the changes experienced in the slot after tooth movement, like scanner electron microscopy which has been used to analyze the slot dimensions and static frictional resistance of stainless steel alloy fixed multibracket appliance [36]. Additionally, computer-aided light microscopy [1, 36], and digital electronic microscopy [6, 13] measurement techniques have been also used to check the slot size and archwire geometries. In addition, digital software has been used to measure the slot; however, none of these techniques have been able to quantify the wear experienced by the slot after the orthodontic treatment [37]. However, Zubizarreta-Macho et al. used the morphometric measurement technique to accurately quantify the area and volume of the remaining cement after removal of fixed multibracket appliances, the area and volume of remaining cement removal, the area and volume of enamel removed after cement removal, and the volume of cement used to adhere fixed multibracket appliance by a repeatable and reproducible measurement technique [38] and Triduo et al. also used the morphometric measurement technique to accurately quantify the area and volume of enamel removed after interproximal enamel reduction [39]. This measurement technique has also been used by Belanche et al. to quantify the cement excess and enamel loss after debonding lingual multibracket appliance therapy [40, 41]. Furthermore, the morphometric measurement technique has been also applied to accurately analyze the influence of drilling technique on the wear of dental implant drills and hence on the osteotomy site preparations after 30 uses [42]. Nevertheless, none of these techniques of measurement previously described have been able to quantify the wear experienced by the bracket slot.

Major et al. reported that the deformations showed at the top surface of the bracket slot at a 63° twist were from 7.0 to 70 µm in stainless steel self-ligating brackets [18]. Moreover, maximum deformations were shown at the mesial and distal top edges of the bracket slot walls, so the warping of the slot walls is not uniform during torque application [18]. Magesh et al. reported a gradual deformation in the bracket slot from the bottom to the top locations, specifically in the gingival slot walls in both stainless steel and ceramic fixed multibracket appliances [1].

The present study selected two operators to analyze the repeatability and reproducibility of the digital measurement technique to quantify the area and volume of wear associated to the bracket slot walls of the fixed multibracket appliance after orthodontic treatment. The addition of more operators will not provide different results, since the Gage R&R statistical analysis is not sensible to the number of operators, since two operators have demonstrated reliability in previous studies [38–41].

## Conclusion

In conclusion, within the limitations of this study, our results showed that the digital measurement technique allowed a repeatable and reproducible measurement technique to quantify the area and volume of wear associated to the bracket slot walls of the fixed multibracket appliance after orthodontic treatment and it can be used for further studies.

## Data Availability

The datasets used and/or analysed during the current study are available from the corresponding author on reasonable request.
